# *NT5E* Mutations That Cause Human Disease Are Associated with Intracellular Mistrafficking of NT5E Protein

**DOI:** 10.1371/journal.pone.0098568

**Published:** 2014-06-02

**Authors:** Michel Fausther, Elise G. Lavoie, Jessica R. Goree, Giulia Baldini, Jonathan A. Dranoff

**Affiliations:** 1 Division of Gastroenterology & Hepatology, University of Arkansas for Medical Sciences, Little Rock, Arkansas, United States of America; 2 Research Service, Central Arkansas VA Healthcare System, Little Rock, Arkansas, United States of America; 3 Department of Biochemistry and Molecular Biology, University of Arkansas for Medical Sciences, Little Rock, Arkansas, United States of America; Massey University, New Zealand

## Abstract

Ecto-5′-nucleotidase/CD73/NT5E, the product of the *NT5E* gene, is the dominant enzyme in the generation of adenosine from degradation of AMP in the extracellular environment. Nonsense (c.662C→A, p.S221X designated F1, c.1609dupA, p.V537fsX7 designated F3) and missense (c.1073G→A, p.C358Y designated F2) *NT5E* gene mutations in three distinct families have been shown recently to cause premature arterial calcification disease in human patients. However, the underlying mechanisms by which loss-of-function NT5E mutations cause human disease are unknown. We hypothesized that human *NT5E* gene mutations cause mistrafficking of the defective proteins within cells, ultimately blocking NT5E catalytic function. To test this hypothesis, plasmids encoding cDNAs of wild type and mutant human NT5E tagged with the fluorescent probe DsRed were generated and used for transfection and heterologous expression in immortalized monkey COS-7 kidney cells that lack native NT5E protein. Enzyme histochemistry and Malachite green assays were performed to assess the biochemical activities of wild type and mutant fusion NT5E proteins. Subcellular trafficking of fusion NT5E proteins was monitored by confocal microscopy and western blot analysis of fractionated cell constituents. All 3 F1, F2, and F3 mutations result in a protein with significantly reduced trafficking to the plasma membrane and reduced ER retention as compared to wild type protein. Confocal immunofluorescence demonstrates vesicles containing DsRed-tagged NT5E proteins (F1, F2 and F3) in the cell synthetic apparatus. All 3 mutations resulted in absent NT5E enzymatic activity at the cell surface. In conclusion, three familial NT5E mutations (F1, F2, F3) result in novel trafficking defects associated with human disease. These novel genetic causes of human disease suggest that the syndrome of premature arterial calcification due to NT5E mutations may also involve a novel “trafficking-opathy”.

## Introduction

The *NT5E* gene encodes the membrane-associated protein ecto-5′-nucleotidase/CD73/NT5E [EC 3.1.3.5], a 70 kDa, glycosylphosphatidylinositol (GPI)-anchored ectoenzyme and a key component of purinergic signaling pathways [1]. Cell-surface NT5E catalyzes the degradation of the nucleotide AMP to the biologically active nucleoside adenosine, in the extracellular milieu [2]. NT5E ectoenzyme is widely expressed in tissues and is commonly referenced as a differentiation marker for a variety of cell types, including B and T lymphocytes, photoreceptor precursor cells, mesenchymal cells, olfactory microvillar cells, somatic cells, and liver myofibroblasts [3]–[9]. NT5E is known to modulate a number of cellular functions dependent on signaling through four specific G-protein coupled receptors for adenosine, including proliferation, apoptosis, activation [1], [8], [10], but also to act as an adhesion molecule, by regulating migratory functions of normal and cancerous cells [10]–[13]. A recent report by St-Hilaire et al. [14] demonstrated that three family cohorts suffer from premature arterial calcification due to homozygous *NT5E* gene mutations: nonsense mutation (c.662C→A, p.S221X) in exon 3, missense mutation (c.1073G→A, p.C358Y) in exon 5, and nonsense (c.1609dupA, p.V537fsX7) in exon 9 [14]. Indeed, all three *NT5E* mutations were found to be associated with loss of functional enzymatic activity, and cause an imbalance in the metabolic pathway of pyrophosphate in circulation, eventually leading to abnormal vascular calcification. However, the precise molecular mechanisms by which NT5E mutations induce cellular defects have not been identified. Of note, in silico analysis of the potential effects of each mutation on NT5E protein structure indicated that, although all three actually result in NT5E proteins lacking catalytic activity, alterations would affect distinct features of newly-synthesized NT5E proteins such as conformation and/or membrane anchoring (Table 1). Based on these observations, we investigated the cellular fate of mutant NT5E proteins and hypothesized that these mutant proteins do not traffic intracellularly akin to their wild type counterparts. In this report, we generated DsRed fusion proteins as a tool to track wild type and mutant human NT5E proteins by microscopy and immunoblot analysis. Our results show that all 3 mutations in *NT5E* gene lead to synthesis of mutant NT5E proteins with no biochemical activity and aberrant trafficking pathways to plasma membrane, when compared to wild type NT5E protein. Because growing literature now shows that the biological functions of NT5E are not exclusively limited to its ability to generate adenosine, strategies to re-establish normal protein trafficking and protein tethering to the plasma membrane may have therapeutic potential to restore related functions in patients carrying *NT5E* mutations.

**Table 1:** Predicted features of wild type and mutant human DsRed-NT5E fusion proteins.

DsRed-hNT5E protein	Amino acid length§	Isoelectric point§	Molecular weight (in Daltons)§	Peptide signal¶	Thrombin cleavage site¶	C-terminal GPI anchor
**Wild type**	825/797	6.06/5.95	91360.1/88657.8	1:27	1:273	Yes
**F1**	471/443	5.61/5.46	51905.0/49202.7	1:27	1.273	No
**F2**	825/797	6.06/5.95	91420.2/88717.9	1:27	1.273	Yes
**F3**	793/765	5.94/5.83	87666.7/84964.4	1:27	1:273	No

§ Before/after maturation.

¶ Total number/position of residue(s).

In silico analysis of wild type and mutant DsRed-tagged human NT5E (DsRed-hNT5E) fusion protein sequences was performed using various bioinformatics tools: ExPASy ProtParam tool for amino acid length, isoelectric point, molecular weight [49]; ExPASy SignalP 4.0 tool for peptide signal location identification [50]; ExPASy PeptideCutter tool for thrombin cleavage site identification [49]; and GPI-SOM tool for C-terminal GPI anchor site recognition [51]. Note: number/position of residues may differ from those of native human NT5E protein.

## Results

We generated expression vectors encoding cDNAs for wild type and mutant human NT5E (hNT5E) proteins tagged with red DsRed monomer fluorescent probe (see “*Materials and Methods*” section). This procedure was essential in allowing us to label and track both wild type and mutant hNT5E proteins, and avoid any potential effect of mutations on hNT5E antigenicity. The design of DsRed-fusion constructs took the following into account: 1) hNT5E is subject to signal peptide removal [15]; 2) NT5E is anchored to plasma membrane via a glycosylphosphatidylinositol molecule [15]; and 3) a similar approach had been previously used to modify/tag mouse NT5E protein [16]. Hence, the expression vectors were designed to encode cDNA for DsRed-hNT5E fusion proteins consisting of DsRed probe attached to the N-terminal end of wild type and mutant hNT5E mature form [17]. Protein sequences of DsRed-hNT5E proteins were analyzed using bioinformatics programs to verify preservation of key features of unlabeled wild type hNT5E protein sequence (Table 1). Afterwards, we performed validation experiments (immunofluorescence, immunoblot and enzyme histochemistry) using expression vectors for DsRed-hNT5E fusion proteins for transient transfection in COS-7 cells that are devoid of any detectable ectonucleotidase activity [18]. Thus, all findings of NT5E protein expression or activity represent only exogenously and transiently expressed proteins in the model COS-7 cell line used here.

### Generation and validation of DsRed molecular labeling of wild type and mutant hNT5E proteins

**Figure 1:**
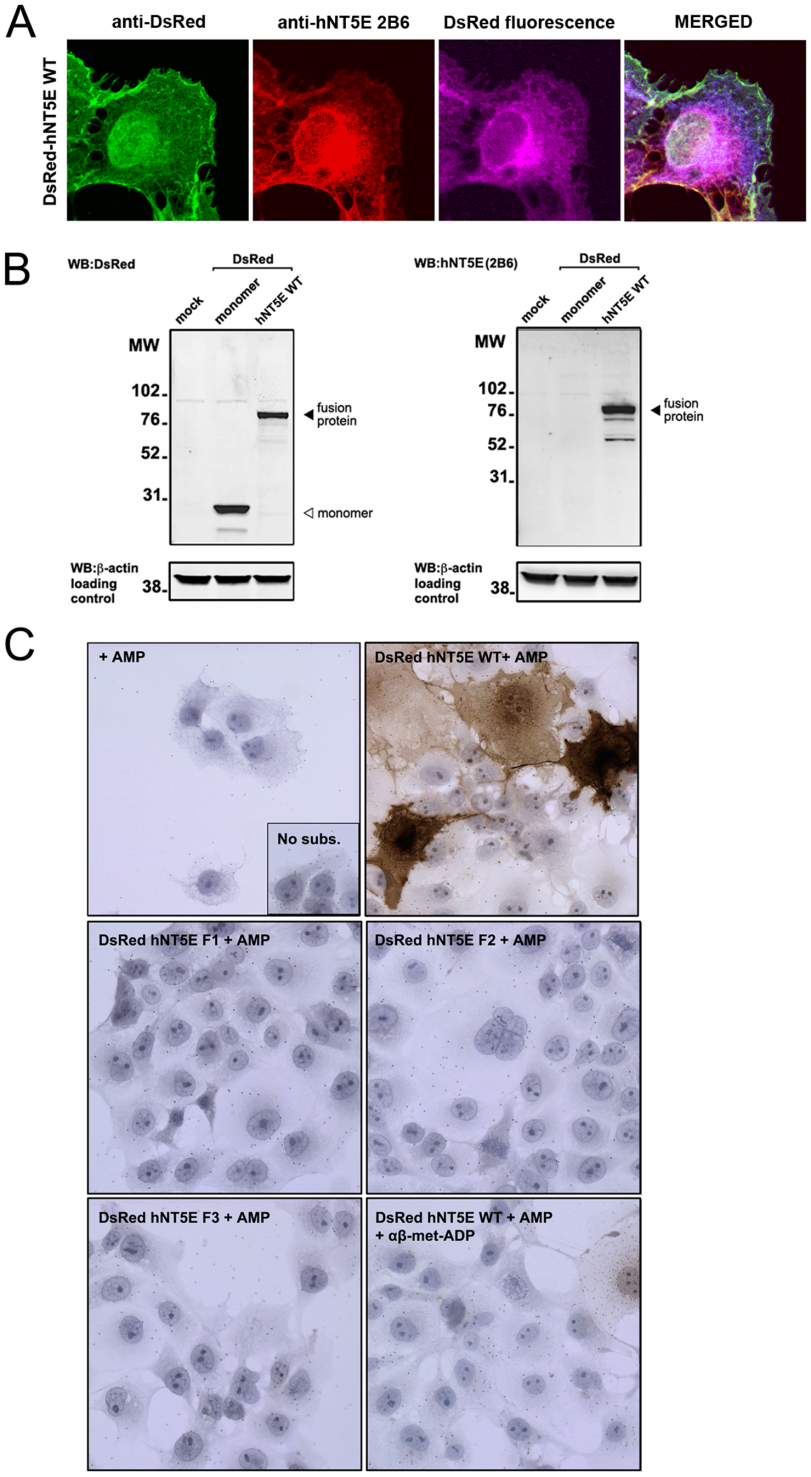
Characterization of DsRed-NT5E fusion proteins. A. COS-7 cells were transiently transfected with an expression vector for DsRed fused to wild type form of human NT5E (DsRed-hNT5E WT) and fusion proteins were localized by immunofluorescence with antibodies against fluorescent DsRed (green) and native hNT5E (red) proteins, and by internal fluorescence (purple). The merged picture shows obvious overlap of all 3 signals indicating that DsRed-hNT5E fusion protein is intact and contains both antigens. *Magnification 400X*. B. Immunoblot analysis of total protein extracts from mock-transfected (mock) or transfected COS-7 cells with expression vectors for DsRed monomer and DsRed-hNT5E (DsRed NT5E) fusion protein using DsRed (left) and hNT5E (right) antibodies. Both antibodies detect a protein with a molecular weight of approximately 90 kiloDaltons (black arrowheads, left and right) while DsRed antibody only detects the DsRed monomer (white arrowhead, left) indicating that DsRed-hNT5E fusion is intact and contains both antigens. Total β-actin protein was used as loading control. C. Enzyme histochemistry of mock-transfected or transfected COS-7 cells with expression vectors for DsRed-hNT5E wild type (WT) and mutant (F1–3) proteins using adenosine monophosphate (AMP) as substrate. Only COS–7 cells transfected with the expression vector for DsRed-hNT5E wild type fusion protein displayed AMPase activity, which could be blocked in presence of known NT5E inhibitor α/β-methylene-ADP. Mock-transfected or COS-7 cells transfected with expression vectors for DsRed-hNT5E mutant (F1–3) proteins lack AMPase activity. *Magnification 200X*.

In COS-7 cells transiently transfected with wild type DsRed-hNT5E vector, double immunofluorescence staining (Figure 1A) with polyclonal anti-DsRed antibody (Figure 1A, *anti-DsRed*, green) and monoclonal anti-hNT5E clone 2B6 (Figure 1A, *anti-hNT5E 2B6*, red) clearly co-localized with intrinsic DsRed fluorescence (pseudo-colored in purple, *DsRed* and *MERGED*). We next extracted total proteins from the same transfected cells and analyzed their contents by immunoblot in wild type DsRed-hNT5E fusion proteins. In these experiments, we used COS-7 cells transiently transfected with DsRed monomer expression vector, and mock-transfected COS-7 cells as controls for antibody specificity. Immunoblot analysis with anti-DsRed antibody (Figure 1B, left panel) detected in the sample corresponding to COS-7 cells overexpressing DsRed monomer a single band with a molecular weight around 28 kDa corresponding to DsRed monomer proteins (Figure 1B, left panel, *DsRed monomer*, 2^nd^ lane). The same anti-DsRed antibody also detected in the sample corresponding to COS-7 cells overexpressing DsRed-hNT5E a single band, with a molecular weight around 90 kDa (Figure 1B, left panel, *DsRed hNT5E WT*, 3^rd^ lane). That molecular weight value would closely correspond to the predicted molecular weight of DsRed-hNT5E wild type fusion protein after maturation (Table 1) and is greater than the one of native hNT5E protein (data not shown). As expected, no band was detected in mock-transfected COS-7 cells. Immunoblotting analysis with anti-hNT5E clone 2B6 (Figure 1B, right panel) did not show reactivity in DsRed monomer-transfected COS-7 cells sample (Figure 1B, right panel, *DsRed monomer*, 2^nd^ lane). In contrast, the same anti-hNT5E clone 2B6 antibody detected a major band in the sample corresponding to DsRed-hNT5E-overexpressing COS-7 cells with a molecular weight around 90 kDa (Figure 1B, right panel, *DsRed hNT5E WT*, 3^rd^ lane), as it was observed with anti-DsRed immunoblot (Figure 1B, left panel, *DsRed hNT5E WT*, 3^rd^ lane). As expected, again, no band was detected in mock-transfected COS-7 cells. We finally tested the biochemical AMPase activity at the cell surface of intact COS-7 cells overexpressing DsRed-hNT5E fusion proteins by enzyme immunohistochemistry, using adenosine monophosphate (AMP) substrate (Figure 1C). Brown precipitates indicative of inorganic phosphate release and AMPase activity in the extracellular milieu were only observed upon incubation with AMP substrate, in COS-7 cells transfected with the expression vector for DsRed-hNT5E wild type fusion protein (Figure 1C, *COS-7 DsRed hNT5E WT + AMP*). Such activity could be blocked in these cells when specific NT5E inhibitor α/β-methylene-ADP was added to the incubation medium. Similar results were obtained with the expression vector for wild type hNT5E protein (data not shown). As expected, mock-transfected COS-7 cells as well as COS-7 cells transfected with expression vectors for mutant DsRed-hNT5E F1-3 fusion proteins did not exhibit any AMPase activity (Figure 1C, *COS-7 DsRed hNT5E F1-3 + AMP*). Taken together, these results show that addition of red fluorescent DsRed monomer probe does not affect the expression, distribution and AMPase activity of wild type hNT5E mature protein, therefore validating use of DsRed-hNT5E-encoding expression vectors for tracking studies.

### Subcellular distribution of DsRed-hNT5E fusion proteins in transiently-transfected COS-7 cells - Confocal microscopy studies

**Figure 2:**
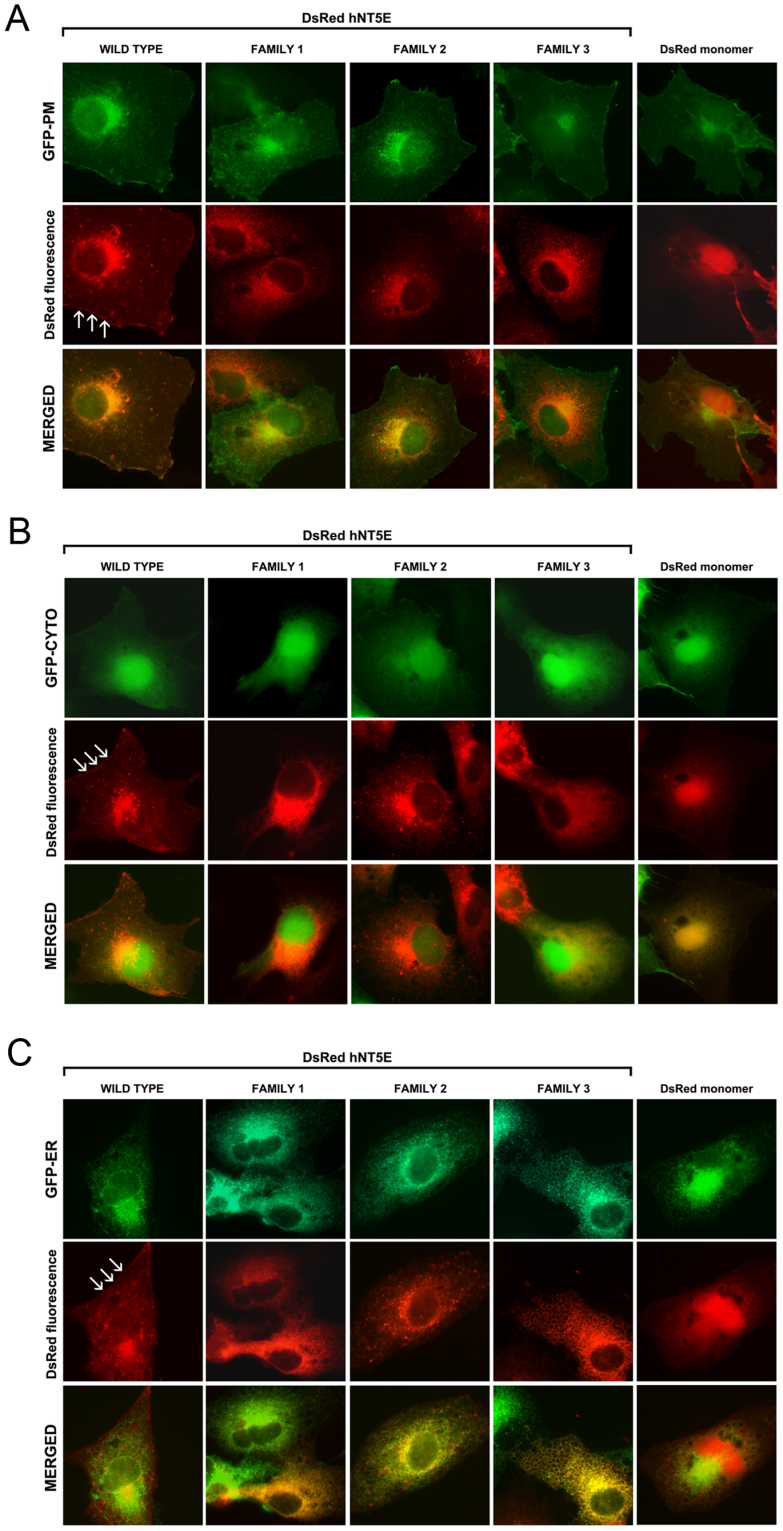
Subcellular distribution of DsRed-NT5E fusion proteins. COS-7 cells were transiently transfected with expression vectors for DsRed monomer, wild type (WT) and mutant (F1–3) DsRed-hNT5E proteins and labeled with GFP-based stains specific for subcellular compartments: plasma membrane (*A, GFP-PM*), cytoplasm (*B, GFP-CYTO*) and endoplasmic reticulum (*C, GFP-ER*). A. While punctate fluorescence signals for all DsRed-hNT5E fusion proteins are observed in areas surrounding nuclei, only DsRed-hNT5E wild type form is also detected at the plasma membrane level and co-localizes with the plasma membrane-specific GFP stain (arrows). Fluorescence signal for control DsRed monomer was more uniformly distributed within cells relatively to DsRed-hNT5E fusion proteins and absent from the plasma membrane compartment. *Magnification 1000X*. B. All DsRed-hNT5E fusion proteins decorate intracellular punctate-shaped structures, with the exception DsRed-hNT5E wild type form being also present at the plasma membrane level (arrows). Also, fluorescence signal for DsRed-hNT5E F3 fusion protein appears to partially co-localize with the cytoplasm-specific GFP stain. Fluorescence signal for control DsRed monomer completely overlaps with that of the cytoplasm-specific GFP stain. *Magnification 1000X*. C. Fluorescence signals for all three mutant DsRed-hNT5E fusion proteins significantly co-localize with that of endoplasmic reticulum-specific GFP stain, although DsRed-hNT5E F2 fusion protein to a lesser extent than its F1 and F3 counterparts. Fluorescence signal for DsRed-hNT5E wild type form only partially co-localizes with that of endoplasmic reticulum-specific GFP stain, as it is also noted for control DsRed monomer. DsRed-hNT5E wild type protein is the only fusion protein associated with the plasma membrane (arrows) *Magnification 1000X*.

COS-7 cells transiently expressing wild type and mutant human DsRed-hNT5E (red signal) were labeled with GFP fusion proteins (green signal) specifically targeted to plasma membrane, cytoplasm and endoplasmic reticulum compartments. In transfected cells labeled with plasma membrane-targeting GFP fusion protein (GFP-PM), only fluorescence signal for DsRed-hNT5E wild type fusion protein was observed at the level of the plasma membrane, predominantly co-localizing (yellow-orange signal) with the one for GFP-PM (Figure 2A, *WILD TYPE-MERGED*, arrows), and to less extent within the cytoplasm, exhibiting a distinctive punctate distribution pattern. In contrast, DsRed-hNT5E F1–3 fusion proteins were primarily observed within the cytoplasm, decorating vesicular-like structures (Figure 2A, *Family1-3-MERGED*). This localization of mutant DsRed-hNT5E proteins differed from DsRed monomer distribution, which was also observed in the cytoplasm but was more evenly scattered (Figure 2A, *DsRed monomer-MERGED*). Most importantly, DsRed-hNT5E F1-3 fusion proteins and DsRed monomer did not significantly overlap with the one of GFP-PM marker at the level of plasma membrane. In transfected cells labeled with cytoplasm-targeting GFP fusion protein (GFP-CYTO), DsRed-hNT5E wild type fusion protein was again observed lining the plasma membrane and following a clear punctate distribution pattern within the cytoplasm (Figure 2B, *WILD TYPE-MERGED*, arrows). This localization was clearly distinct from the one of GFP-CYTO marker. A similar conclusion was drawn after analysis of DsRed-hNT5E F2 fluorescence signal (Figure 2B, *Family2-MERGED*). DsRed-hNT5E F1 and F3 fluorescence signals did partially co-localize with GFP-CYTO marker (Figure 2B, *Family1;3-MERGED*). DsRed monomer fluorescence signal was not observed at the plasma membrane level, but was uniformly distributed within cytoplasm, overlapping significantly with the GFP-CYTO marker (Figure 2B, *DsRed monomer-MERGED*). In transfected cells labeled with endoplasmic reticulum-targeting GFP fusion protein (GFP-ER), DsRed-hNT5E wild type fusion protein was mainly detected at the plasma membrane level and only partially co-localized with the GFP-ER marker (Figure 2C, *WILD TYPE-MERGED*, arrows). While absent from the plasma membrane, DsRed-hNT5E F2 fluorescence signal also partially overlapped with the one of GFP-ER marker (Figure 2B, *Family2-MERGED*). In contrast, DsRed-hNT5E F1 and F3 proteins significantly co-localized with the one of GFP-ER marker and were detected only within the cytoplasm (Figure 2C, *Family1;3-MERGED*). Finally, DsRed monomer fluorescence signal was again absent from the plasma membrane, and detected within the cytoplasm partially co-localizing with the one of GFP-ER marker (Figure 2C, *DsRed monomer-MERGED*). Taken together, these results suggest that mutant DsRed-hNT5E F1-3 fusion proteins, in addition to be enzymatically inactive, assume intracellular trafficking pathways clearly distinct from the one of DsRed-hNT5E wild type fusion protein. While the DsRed-hNT5E wild type fusion protein is observed within ER structures and cytoplasm to some extent, and mainly at the level of plasma membrane, mutant DsRed-hNT5E fusion proteins are mainly detected within cytoplasm (F1 and F3) and ER vesicles (F1–3) compartments. Notably, subcellular distribution of DsRed-hNT5E F2 fusion protein is comparable to the one of DsRed-hNT5E wild type fusion protein, except for its absence from the plasma membrane.

### Subcellular distribution of DsRed-hNT5E fusion proteins in transiently-transfected COS-7 cells - Immunoblot studies

**Figure 3:**
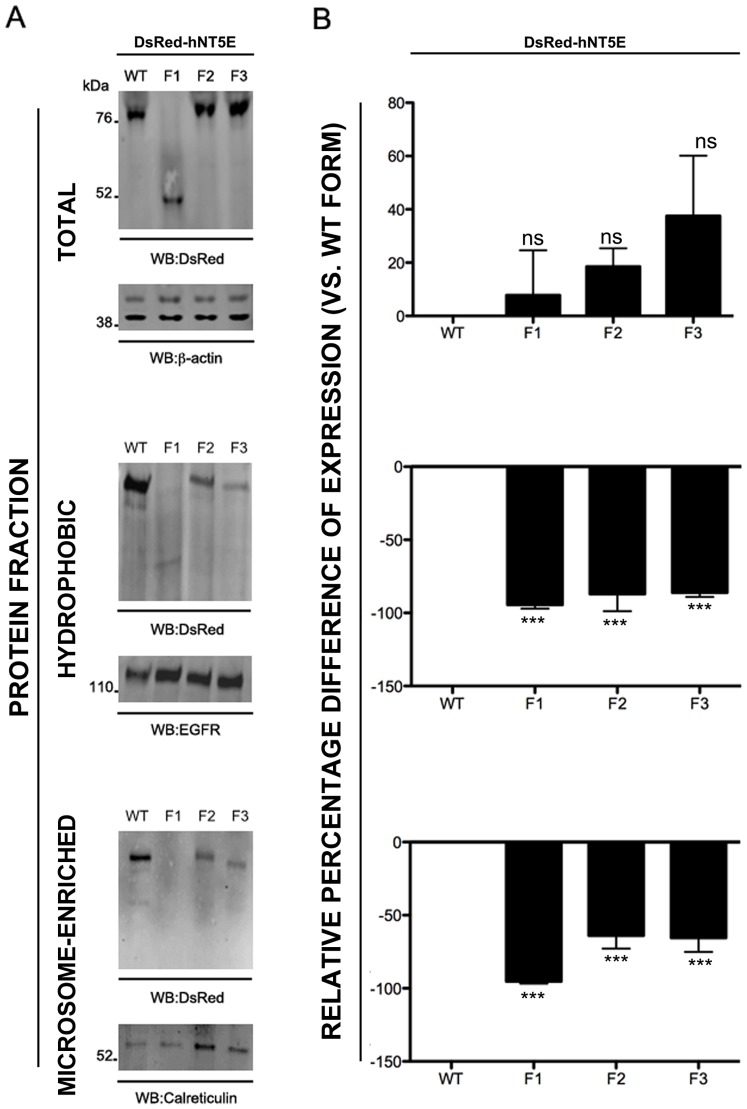
Subcellular compartment expression of DsRed-NT5E fusion proteins. COS-7 cells were transiently transfected with expression vectors for DsRed-hNT5E wild type (WT) and mutant (F1–3) proteins and proteins were fractionated and immunoblot analysis performed as described in “Materials and Methods” section. A. Representative immunoblot analyses of total, hydrophobic and microsome-enriched proteins fractions are depicted (n = 3). For each protein fraction, immunoblot using anti-DsRed (top) and appropriate loading control (bottom) are shown. B. Densitometry analysis of band signal intensity is presented as percentage difference of expression for each DsRed-hNT5E fusion protein, using DsRed-hNT5E wild type fusion protein as baseline. All DsRed-hNT5E fusion proteins are relatively expressed to same extent in transfected cells (DsRed-hNT5E F1, -F2 and F3 vs. DsRed-hNT5E WT, *p* value = 0.1698, not significant, ns, n = 3). Only DsRed-hNT5E wild type fusion protein is abundantly present in hydrophobic protein fraction (DsRed-hNT5E F1, -F2 and F3 vs. DsRed-hNT5E WT; *p* value<0.0001, ***, n = 3). DsRed-hNT5E is more abundant in the microsome-enriched protein fraction, when compared DsRed-hNT5E F1–3 fusion proteins with F1 mutant completely absent (DsRed-hNT5E F1, -F2 and F3 vs. DsRed-hNT5E WT; *p* value<0.0001, ***, n = 3).

Total, hydrophobic, and microsome-enriched protein fractions were extracted from transiently-transfected COS-7 cells and analyzed for their contents in DsRed-hNT5E fusion proteins. Analysis of the total protein fraction reveals that DsRed-hNT5E wild type and mutant fusion proteins are produced to similar levels in COS-7 cells upon transient transfection (Figure 3, *TOTAL*, DsRed-hNT5E F1 7.8%±16.9, -F2 18.5%±6.8 and F3 37.5%±22.6; *p* value = 0.1698). As expected, examination of hydrophobic (plasma membrane-enriched) protein fraction shows that only DsRed-hNT5E wild type protein is abundantly present at the plasma membrane of all DsRed-hNT5E fusion proteins (Figure 3, *HYDROPHOBIC*, DsRed-hNT5E F1 −94.4%±2.6, -F2 −86.9%±11.87 and F3 86.1%±2.9; *p* value<0.0001). Finally, analysis of microsomes-enriched protein fraction shows that DsRed-hNT5E F1–3 fusion proteins are significantly less abundant than their wild type counterpart, with DsRed-hNT5E F1 fusion protein being almost absent (Figure 3, *MICROSOME-ENRICHED*, DsRed-hNT5E F1 −95.2%±1.6, -F2 −63.8%±8.9 and -F3 −65.5%±9.5; *p* value<0.0001). Taken together, these results indicate that, although all DsRed-hNT5E fusion proteins are produced roughly to the same extent, only DsRed-hNT5E wild type protein is able to efficiently reach the plasma membrane compartment. These data also suggest that mutant protein trafficking mechanisms are deficient within and beyond the ER compartment as DsRed-hNT5E F1-3 fusion protein contents are greatly reduced in the microsome-enriched fraction, when compared to DsRed-hNT5E wild type protein.

### Biochemical activity of DsRed-hNT5E fusion proteins in transiently-transfected COS-7 cells

**Figure 4:**
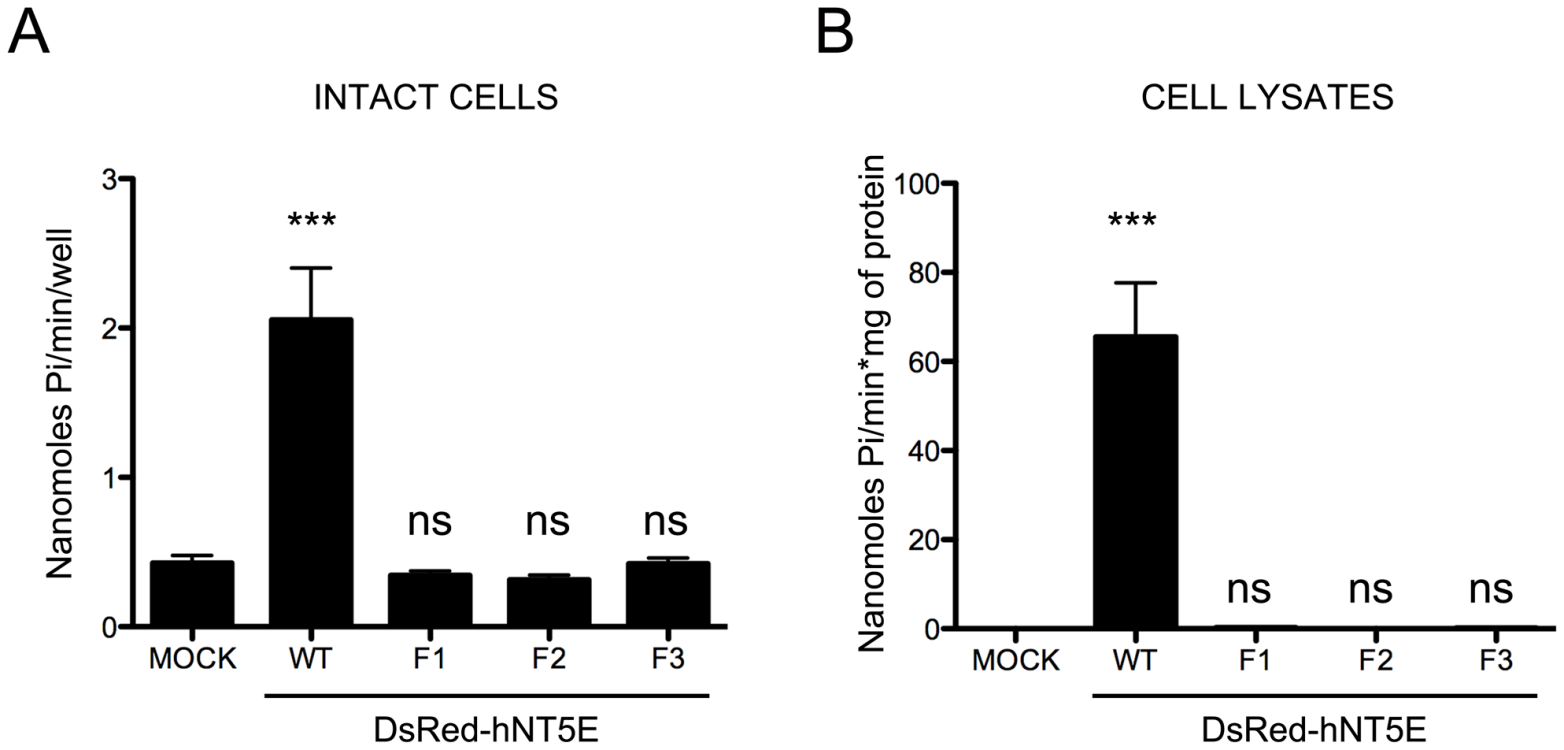
Subcellular compartment expression of DsRed-NT5E fusion proteins. Intact or protein extracts from COS-7 cells transiently transfected with expression vectors for wild type (WT) and mutant (F1–3) DsRed-hNT5E fusion proteins were assayed for AMPase activity as described in “Materials and Methods” section. A. Surface AMPase activity is only found in transfected cells transiently expressing DsRed-hNT5E wild type fusion protein (DsRed-hNT5E WT vs. MOCK; *p* value<0.0001, n = 3). No AMPase activity was detected in mock-transfected cells as expected, or transfected cells transiently expressing DsRed-hNT5E F1-3 proteins (DsRed-hNT5E F1, -F2, -F3 vs. MOCK; *p* value<0.0001, not significant, ns, n = 3). B. AMPase activity is only measured in protein extracts from transfected cells transiently expressing DsRed-hNT5E wild type fusion protein (DsRed-hNT5E WT vs. MOCK; *p* value<0.0001, significant, ***, n = 3). Mock-transfected cells or transfected cells transiently expressing DsRed-hNT5E F1–3 proteins lack any measurable AMPase activity (DsRed-hNT5E F1, -F2, -F3 vs. MOCK; *p* value<0.0001, not significant, ns, n = 3).

The ability to degrade AMP nucleotide substrate of DsRed-hNT5E fusion proteins was tested using the Malachite Green assay for detection of released inorganic phosphate. AMPase activity assays were performed on intact cells or using lysates from transiently-transfected COS-7 cells. In both assays, only samples from COS-7 cells transfected with expression vector for DsRed-hNT5E wild type fusion protein exhibited AMP hydrolysis (Figure 4A and 4B, *WT*). Mock-transfected COS-7 cells or COS-7 cells transiently transfected with expression vectors for DsRed-hNT5E F1–3 fusion proteins had no detectable activity (Figure 4A and 4B, *MOCK* and *F1-3*). Transfected COS-7 cells were also incubated in presence of sodium butyrate (NaB), or 4-phenyl sodium butyrate (4PBA) molecules for 24 hours (prior to ending 72-hour transfection reaction) and cell lysates extracted and tested for AMP hydrolysis activity. This experiment was designed to test whether both NaB and 4PBA molecules could be used as molecular chaperones and induce the recovery of biological AMPase activity from mutant DsRed-hNT5E proteins, as it had been previously done for other mutant proteins such as CFTR [19], [20]. When compared to control transfected cells (i.e. grown in normal conditions, Figure 4B), incubation with either NaB or 4PBA molecules had no effect was observed on AMPase activity levels from cell lysates from COS-7 cells transfected with expression vectors for DsRed-hNT5E F1–3 fusion proteins, which were still negligible (not shown).

## Discussion

In the present report, we report here novel trafficking defects caused by *NT5E* genetic mutations in the context of the newly-described hereditary vascular calcification disorder due to these mutations, now shown to cause disease in three distinct family cohorts [14]. We examined the localization and distribution of recombinant DsRed-labeled wild type and mutant human NT5E proteins upon transient transfection in NT5E-deficient COS-7 cells. Our microscopy studies, while confirming that all three mutant F1-3 NT5E proteins exhibit no catalytic AMPase activity, have also shown that these defective proteins are also associated with obvious defects in intracellular trafficking, as compared to their wild type counterparts. Indeed, of all DsRed-NT5E fusion proteins examined, only the wild type form is able to reach the plasma membrane subcellular compartment. These observed trafficking defects might be attributable to predicted alterations in protein structure caused by each mutation studied (Table 1). For F1 (c.662C→A, p.S221X) and F3 (c.1609dupA, p.V537fsX7), nonsense mutations resulting respectively in truncated proteins and in truncated proteins with a non-functional/absent GPI anchor motif (Table 1), such an observation was not utterly surprising. However, in the case of F2 (c.1073G→A, p.C358Y) missense mutation, the absence of mutant protein at the cell surface was unexpected, because the mutation was anticipated to likely affect the enzyme ability to degrade AMP substrate, but not its normal synthesis process and intracellular transport within cells. Moreover, our in silico analysis of DsRed-hNT5E F2 protein further reveals that the GPI anchor motif should be preserved (Table 1). The microscopy observations were also confirmed by immunoblot studies showing that, although both DsRed-hNT5E wild type and mutant proteins are produced to similar levels in total protein samples, the wild type protein is the only form detected in the hydrophobic (plasma membrane) protein fraction and thus reaching the cell surface. Moreover, immunoblot analysis of the microsome-enriched protein fraction indicates that F1 protein contents are negligible while F2 and F3 protein contents are clearly detectable but greatly reduced when compared to the wild type form. When reconciled with our microscopy observations, this may suggest that all mutant proteins alike the wild type NT5E protein transit through the ER compartment, but only for a very short period of time (F1 mutant protein) or associated with an inefficient synthesis and maturation process (F2 and F3 mutant proteins). In the case of F3 mutation, although this hypothesis was not tested here, it is possible that the mutation may render the nascent polypeptides resistant to processing by GPI transamidase enzyme, a critical step for addition of GPI anchor molecules [21]. Improper GPI anchoring could lead to either secretion (less likely, but possible) or sequestration of F3 mutant proteins within ER compartment (or beyond), and eventually degradation. Alternatively, improper protein folding resulting from all three mutations could provide an explanation for the observations made here, as it has been demonstrated for mutations of human tissue-non-specific alkaline phosphatase gene [22], [23]. The effect of commonly-used molecular chaperones sodium butyrate and its analogue sodium-4-phenyl butyrate was also tested on biochemical activity of DsRed-hNT5E mutant proteins, based on previous activity rescue studies on transmembrane F508del CFTR mutant proteins [24]. The AMPase activity of DsRed-hNT5E F1-3 mutant proteins could not be rescued and remained at the level of mock control cells, when transfected COS-7 cells were incubated with each of these compounds at a concentration of 5 mM for 24 hrs before assay. One possible explanation for the lack of chaperone efficacy of both compounds toward mutants could be the concentration tested in our biochemical assays. Even though concentrations higher than 5 mM have been used previously in rescue studies, we used a concentration that had been tested before and could be tolerated by COS-7 cells without any significant toxic effect [20], [25]. Another explanation could be that both compounds cannot rescue NT5E catalytic activity in mutants simply because these proteins are inactive intrinsically, as potentially a result of inherent conformation alterations or inability to form enzymatically-active dimers. The case can certainly be made for F1 mutant proteins, which are shortened NT5E proteins partially lacking the signature C-terminal domain conserved in all 5′nucleotidase proteins. Of note, F508del CFTR mutant proteins retain some level of biological activity (although relatively reduced compared to wild type proteins) [24].

Importantly, caution is required when interpreting data presented here and making general assumptions with regard to CD73 cellular dynamics and contribution to cell functions. Because of the chosen heterologous over-expression system, all DsRed-NT5E fusion proteins were produced at similar mRNA (not shown) and protein (Figure 3) levels. This contrasts with data obtained from patient studies, in which described F1 mutation was associated with overall decreased gene expression [14]. Also, it is certainly hard to predict whether the intracellular trafficking defects described here in transiently-transfected COS-7 cells would occur in other cell types, and if so, to the same extent. Indeed, it has been previously reported that NT5E ectoenzyme distribution between intracellular compartments can vary depending on the cell type studied [26].

What could be the functional consequences of *NT5E* mutations? On one hand, besides the impact on pyrophosphate balance in the circulation described in the original article [14], it is anticipated that all three mutations will affect regulation of extracellular adenosine levels and related pathways [2], [10], [14], [27], [28], resulting from loss of enzymatic activity which is an established feature in all mutant proteins. On the other hand, it is well possible that all three NT5E mutations, by affecting NT5E protein targeting at the cell surface, may impact its function as an adhesion molecule [29], to mediate cell-to-cell [11], [12] and cell-to-matrix interactions [30]. For instance, cell-surface NT5E is a key regulator of extravasation process in immune cells [11], and adhesion/migration/invasion properties of various cancer cell lines [13], [31]. Moreover, recent studies suggest that effectiveness of antibody-based NT5E cancer therapy owes not only to adenosine signals inhibition in the metastasis microenvironment [27], [32], [33], but also to suppression of circulating tumor cells ability to extravasate and colonize tissues following NT5E clustering and subsequent internalization [34].Therefore, it can be hypothesized that cell adhesion mechanisms or functions requiring cell-surface expression of NT5E proteins would likely be affected by the three described *NT5E* mutations. Future work should certainly explore the potential impact of NT5E mutations in other diseases relevant to NT5E (non-)catalytic activity functions such as, organ injury/fibrosis [36]–[39], cell inclusion formation [40], and immunity [28], [41], [42]. Although seemingly counter-intuitive due to the fact that heterozygous patients do not exhibit obvious abnormalities [14], it is nonetheless critical to investigate the occurrence of chimeric proteins and their potential impact of NT5E-dependent functions [35], because NT5E naturally occurs as a dimeric protein [1]. Finally, attempts to recapitulate AMPase activity in patients may require multiples approaches including intravascular delivery of exogenous NT5E activity (F1 mutation) [43], effective molecular strategy to restore GPI anchoring (F3 mutation) or use of compounds that promote premature termination codon read-through (F1 and F3 mutations) [44]. It is important to note that discovery of monogenic diseases is increasingly rare, but critical to the progress of biomedical research [45]. It is thus essential to define the molecular mechanisms, by which monogenic defects exert their adverse effects, both to generate effective models for the understanding of cellular dysfunction and ultimately to identify rational therapeutic approaches.

## Materials and Methods

### Cloning

Standard PCR-based molecular cloning methods and a commercial pDsRed-Monomer-C In-Fusion Ready Vector (Clontech, Mountain View, CA) were used to generate mammalian expression vectors encoding a fusion protein consisting of the DsRed fluorescent probe attached to the N-terminal end of the mature form of human NT5E (hNT5E) protein (NCBI Protein ID: NP_002517.1[27–549]), after peptide signal (NP_002517.1[1]–[26]) cleavage [15]. Plasmids encoding published wild type (1) and mutant (3) hNT5E protein cDNAs (kindly provided by Dr. C. St-Hilaire, NHLBI, Washington, D.C.) were used as PCR templates [14]. PCR amplifications were done with Expand High-Fidelity Plus PCR System (Roche Biosciences, Palo Alto, CA) for maximal elongation fidelity. Ligation reactions were done with T4 DNA Ligase (New England Biolabs, Beverly, MA) for optimal ligation efficiency. Chemically-competent OneShot TOP10 bacteria cells (Invitrogen, Carlsbad, CA) were used for superior transformation efficiency. All constructs were designed so as to insert DsRed protein and consensus thrombin recognition peptide (to allow removal of DsRed tag) sequences between the 26^th^ and 27^th^ amino acids (aa) of hNT5E protein sequence (NP_002517.1[26]–[27]), respectively corresponding to the 78^th^ and 79^th^ base pairs (bp) of human *NT5E* consensus coding DNA sequence (NCBI Nucleotide ID: NM_002526.3[78–79]). In a first step, the pDsRed-Monomer-C vector was modified by directionally ligating a synthetic DNA polylinker containing a single Spe1 restriction (enzyme cutting) site into Sal1/HindIII restriction sites. In a second step, the signal peptide sequence of hNT5E protein (NP_002517.1[1]–[26]) was added upstream of the DsRed-Monomer sequence contained in the pDsRed-Monomer-C vector. Two irrelevant -TT- base pairs were added to the 3′end of the hNT5E signal peptide sequence for maintenance of the correct open reading frame by high fidelity PCR. Nhe1 restriction sites were subsequently introduced at both ends of the resulting 80 bp-long sequence by high fidelity PCR using the following primers:

*Nhe1*: forward, -ATATGCTAGCATGTGTCCCCGAGCCGCGCG-;

*Nhe1*: reverse -ATATGCTAGCAAGGCGCCAGCCGCAGGCCA- (underlined, Nhe1 sites).

The obtained PCR product was ligated into pCRII TOPO vector (Invitrogen) and ligation products were used for transformation bacteria following manufacturer's instructions. Plasmid DNA isolated from at least 3 individual colonies was analyzed by automated sequencing (UAMS DNA Sequencing Core Facility). The plasmid DNA from one selected clone was then Nhe1-digested, purified and ligated into the single Nhe1 restriction site of pDsRed-Monomer-C vector (located 16 bp 5′ upstream of in-frame DsRed-Monomer sequence). The ligation products were used for bacteria transformation, and plasmid DNA isolated from at least 3 individual colonies was analyzed by automated sequencing. This step was carried out only once as nucleotide sequence analysis (EMBL-EBI Clustal Omega bioinformatics software, Sievers et al. 2011) shows full (100%) homology between the wild type and mutant sequences. In a third and final step, a consensus thrombin recognition motif was added to the 5′end of remaining portion of the hNT5E protein sequence (NP_002517.1[27–574]). A single Sal1 restriction site, a thrombin recognition sequence -*CTGGTGCCCAGGGGCAGC*-, an internal EcoR1 restriction site (5′end) and a single Spe1 restriction site (3′end) were added to the corresponding 1647 bp-long coding hNT5E sequence (NM_002526.3[79–1725]) by high fidelity PCR using hNT5E cDNA as template and the following primers

*Sal1In*: forward, -CAGGGGCAGCGAATTCTGGGAGCTTACGATTTTGC-;

*Spe1*: reverse, -ATATTACTAGTCTATTGGTATAAAACAAAGATCAC- (underlined, Spe1 site).

An aliquot (1/10) of the obtained PCR product was then used as template for a nested high fidelity PCR reaction using the following primers

*Sal1Out*: -TTAAGGCCTCTGTCGAC *CTGGTGCCCAGGGGCAGC*- (underlined, Sal1 site; *italic*, thrombin consensus sequence);

*Spe1*: reverse, -ATATTACTAGTCTATTGGTATAAAACAAAGATCAC- (underlined, Spe1 site). The obtained nested PCR product was ligated into pCRII TOPO vector (Invitrogen). The ligation products were used for bacteria transformation and plasmid DNA isolated from at least 3 individual colonies analyzed by automated sequencing. The plasmid DNA from one selected clone was then was Sal1- and Spe1-digested, purified and directionally ligated into Sal1/Spe1restriction sites of pDsRed-Monomer-C vector. This step was carried out for each wild type and 3 mutant cDNAs as nucleotide sequence analysis shows expected variations between the wild type and mutant sequences [14]. Fidelity of all fusion constructs was verified again by automated sequencing. The pDsRed-Monomer-C vector encoding DsRed cDNA alone was used as a control for appropriate experiments.

### Cell line and transfection

African green monkey COS-7 cells (ATCC CRL-1651) were cultured in Dulbecco's Modified Eagle Medium (DMEM) with 10% fetal bovine serum and 1% penicillin-streptomycin (Invitrogen). Transient transfection of COS-7 cells using plasmids encoding cDNAs for DsRed-hNT5E wild type and mutant forms was carried out with Lipofectamine 2000 reagent (Invitrogen) for 72 hrs, according to the manufacturer's instructions. Where appropriate, protein concentration was estimated using the Microplate BCA Protein Assay Kit (Pierce Biotechnology, Rockford, IL) with reference to bovine serum albumin (BSA) as a protein standard. For enzymatic activity assays on protein extracts, cells were incubated in absence or presence of 5 mM sodium butyrate (Sigma, St Louis, MO) or 5 mM 4-phenyl sodium butyrate (Sigma) 48 hrs post-transfection for additional 24 hrs. For enzymatic activity assays on intact cells, cells were split 24 hrs post-transfection, plated in 24-plate wells and cultured for additional 48 hrs. For fluorescence microscopy, cells were split and plated on sterile coverslips (10^5^/coverslip) 24 hrs post-transfection and cultured for additional 48 hrs. For each assay, experiments were performed 3 times, except for enzymatic assays with sodium butyrate and 4-phenyl sodium compounds 2 times. The COS-7 cell line was used in this study because it exhibits negligible basal levels of nucleotide-metabolizing enzyme activities [18].

### Enzyme histochemistry and Immunofluorescence microscopy

Transfected COS-7 cells were fixed in freshly prepared 4% (wt/vol) paraformaldehyde in neutral PBS (pH 7.2) for 20 min at room temperature. For enzyme histochemistry experiments, hNT5E AMPase activity was localized using a modified Wachstein/Meisel lead phosphate method, as previously described [46] The enzymatic reaction was carried out, by incubating transfected cells with 200 µM adenosine monophosphate (AMP) nucleotide (Sigma) as substrate for 2 hrs at 37°Celsius. In a separate set of experiments, 1 mM α/β-methylene-ADP (Sigma) was used as a hNT5E inhibitor. Control assays were performed in the absence of nucleotide. Cell nuclei were then counterstained with aqueous haematoxylin (Sigma) and Mowiol4–88 liquid mountant (EMD Millipore, Billerica, MA) directly applied on coverslips. Specimens were examined at 200× magnification by bright field imaging with an Olympus BX51 microscope. For fluorescence microscopy, protein co-localization experiments were conducted as follows: green fluorescent protein-based CellLights BacMam 2.0 reagents (Molecular Probes, Eugene, OR) specifically labeling cytoplasm, plasma membrane and endoplasmic reticulum cell compartments were added to transfected cells on 48 hrs post-transfection and incubated for additional 24 hrs. Cells were washed and fixed (as described above), and ProLong Gold Reagent liquid mountant (Molecular Probes) directly applied on coverslips. Specimens were examined at 1000× magnification using Zeiss Axiovert ImagerZ1. In a separate set of experiments, fixed transfected cells were co-incubated with rabbit polyclonal anti-DsRed (cat#632496, Clontech) and mouse monoclonal anti-hNT5E (clone 2B6, cat#MCA4622GA, AbD-Serotec, Raleigh, NC) antibodies overnight at 4°Celsius. Coverslips were then incubated with appropriate goat Alexa Fluor-conjugated anti-rabbit IgG and anti-mouse IgG antibodies (Molecular Probes) 1 h at room temperature. ProLong Gold Reagent liquid mountant was subsequently applied on coverslips. Specimens were examined using a confocal Zeiss LSM 510 META microscope equipped with Krypton/Argon, Diode, and Helium/Neon lasers at 400× magnification. Triple-labeled specimens were serially excited at 488 nm and observed at >515 nm to detect Alexa Fluor 488, excited at 561 nm and observed at >595 nm to detect DsRed using the Diode laser, and then excited at 633 nm and observed at >650 nm to detect Alexa Fluor 647 using the He/Ne laser.

### AMPase activity assays

Protein extracts from transfected COS-7 cells were prepared, as previously described [47]. Ectonucleotidase activity of protein extracts from transfected cells and, of intact COS-7 transfected cells was determined by measuring the liberated inorganic phosphate (Pi) from degradation of AMP nucleotide substrate according to the Baykov method [48], as previously described [47].

### Immunoblot

Following transfection (72 hrs), cells were scraped and pelleted (500×*g* for 5 min) in cold 1X PBS supplemented with protease inhibitors cocktail (Halt Protease Inhibitor Single Use Cocktail, Pierce Biotechnology) before preparation of various protein fractions. Protein samples were prepared from the following cell compartments: total proteins using a mild-strength lysis buffer (25 mM Tris-hydrochloride, 50 mM sodium chloride, 1 mM phenylmethanesulfonyl fluoride, 1% IGEPAL CA-930), hydrophobic (plasma membrane-associated) proteins using the MEM-PER Membrane Protein Extraction kit (Pierce Biotechnology), and crude microsomal fraction proteins using the Endoplasmic Reticulum Isolation Kit (Sigma). Protein fractions were separated by SDS-PAGE under reducing conditions, and transferred onto a polyvinylidene difluoride membrane (Immobilon/Millipore, Bedford, MA). Membranes were blocked with 1X Tris-Buffer Saline, 5% milk, 0.1% Tween-20 and incubated with rabbit polyclonal anti-DsRed antibody (Clontech) followed by fluorescent-labeled goat anti-rabbit IgG antibody, or mouse monoclonal anti-hNT5E antibody (clone 2B6) followed by fluorescent-labeled goat anti-mouse IgG antibody, and bands were visualized by use of a Typhoon 9400 imaging system (GE Healthcare Life Sciences, Pittsburgh, PA). Membranes were subsequently stripped and incubated with anti-calreticulin (cat#ADI-SPA-600-F, Enzo Life Sciences, Farmingdale, NY), and anti-EGFR (cat#PA1-1110, Pierce Biotechnology) antibodies, and mouse monoclonal anti-beta actin clone AC-15 (cat#A5441, Sigma), followed by fluorescent-labeled goat anti-rabbit IgG or anti-mouse IgG antibodies, and band intensity signals were visualized and quantified by use of a GE Typhoon imaging system. Band densitometry analysis was performed using GE Image Quant TL software, version 7.0.

### Statistical analysis

One-way ANOVA with Bonferroni's post-test was performed using GraphPad Prism version 5.0d for Mac, GraphPad Software, San Diego California USA, www.graphpad.com.
