# Correction: *NT5E* Mutations That Cause Human Disease Are Associated with Intracellular Mistrafficking of NT5E Protein

**DOI:** 10.1371/journal.pone.0118252

**Published:** 2015-03-05

**Authors:** The PLOS ONE Staff

The following information is missing from the Funding section: Individual support was provided to MF in the form of a Gilead Sciences Research Scholars Program in Liver Disease Award.
